# Real-world assessment of current migraine prophylaxis in Egypt: a multicenter national study

**DOI:** 10.1186/s10194-025-02246-2

**Published:** 2026-01-08

**Authors:** Salsabil Abo Al-Azayem, Mona A. F. Nada, Ahmed Dahshan, Mona K. Moawad

**Affiliations:** Department of Neurology, Cairo University, Cairo, Egypt

**Keywords:** Migraine, Prophylactic treatment of migraine, Adherence to treatment, MIDAS, ARMS

## Abstract

**Background & objectives:**

Prophylactic treatment plays a crucial role in reducing the frequency, severity, and duration of migraine attacks. This study, the first to address this issue in Egypt, aimed to study the patterns of prophylactic treatment utilization among individuals with migraine and assess adherence to prescribed regimens.

**Methods:**

A cross-sectional study was conducted on 200 individuals with migraine. Headache was assessed as follows: type of migraine, duration of attacks, frequency of headache per month, disability using the Migraine Disability Assessment Scale (MIDAS) and headache severity using a visual analog scale (VAS), and treatment history. Prophylactic treatments were evaluated regarding type, dose, adherence using Adherence to Refills and Medications Scale (ARMS) and efficacy using monthly migraine days (MMD) as well as adherence predictors were assessed.

**Results:**

In our cohort, the median age was 32 years, with women comprising 70.6% of the cohort. For abortive treatment, paracetamol was the most prescribed medication (42%). Regarding prophylactic treatment, adherence was low, with only 30.4% of patients maintaining adherence. Multivariate regression analysis identified the use of antidepressants as an independent predictor of adherence. 83.3% of patients received monotherapy, with antiepileptic drugs being the most prescribed (40%). Notably, polytherapy regimens demonstrated statistically significant superiority in reducing headache duration, MMD, VAS scores, and MIDAS scores (*P* < 0.001).

**Conclusion:**

Non-adherence to prophylactic medication is prevalent among individuals with migraine in Egypt. The severity of migraine attacks is lower in patients receiving polytherapy regimens; however, using antidepressants as prophylactic treatment of migraine is positively associated with treatment adherence.

**Supplementary Information:**

The online version contains supplementary material available at 10.1186/s10194-025-02246-2.

## Background

Migraine is a prevalent and debilitating neurological disorder that poses challenges not only in its management but also in its prevention [1]. While acute treatment options aim to alleviate symptoms during migraine attacks, prophylactic treatment plays a crucial role in reducing the frequency, severity, and duration of these episodes [2]. Despite the proven efficacy of prophylactic therapies, adherence to these treatments remains a persistent challenge. A growing body of evidence highlights that non-adherence is a major barrier to achieving optimal outcomes in migraine care. Factors such as side effects, complex regimens, and individual barriers related to socioeconomic or cultural contexts contribute to this issue [3]. However, the literature is sparse regarding region-specific factors influencing adherence, particularly in low- and middle-income countries (LMIC) such as Egypt.

Research has examined the use of migraine prophylactic medications among individuals enrolled in medical plans. These studies often rely on pharmaceutical records to provide more accurate data on medication usage; however, they are limited by challenges in accurately identifying all individuals with migraine [4]. Meyers et al. [5] highlight the scarcity of real-world data on migraine epidemiology and current treatment approaches, emphasizing the need for such information. Although prophylactic medications for migraine have been available for many years, studies assessing their real-world efficacy and tolerability remain limited [6].

To address these gaps, this study aims to provide a comprehensive assessment of prophylactic treatment utilization and adherence among individuals diagnosed with migraine in Egypt. In a recently published study, it was found that the prevalence of migraine in Egypt is 20% which means that almost 22 million people in Egypt suffer from migraine [7]. That is way, for the first time in this region, the research will examine adherence levels, identify key barriers, and explore the relationship between adherence and treatment outcomes. The primary outcome of the study was to evaluate adherence to prophylactic migraine medication. The secondary outcomes were to (1) describe the prescribing patterns of prophylactic medications for migraine in Egypt, (2) assess drug-related efficacy and adverse effects, and (3) identify factors associated with treatment discontinuation.

## Methods

### Study population

This cross-sectional study with retrospective element screened 1100 patients presenting with headache and included 200 patients aged 14–65 who met the International Classification of Headache Disorders, third edition (ICHD-3) criteria for migraine [8] and agreed to participate. The patients were recruited from two headache centers in Egypt representing the 2 larger governates: Cairo University Hospital (A tertiary center) and a private headache center in Alexandria, between June 2023 and April 2024. To minimize potential bias, the referring neurologists were blinded to the study’s goals, ensuring that patient referrals were based solely on clinical indications rather than study-related factors.

Patients were excluded if they met any of the following criteria: 1. Diagnosed with headaches other than migraine.2. Exhibited focal neurological deficits. 3. Had other neurological diseases.

### Data collection

Demographic and clinical data were collected through face-to-face interviews. The data gathered included: Age at onset, Disease duration, Type of migraine (with or without aura), Type of aura (if present), Duration of attacks (in hours), Frequency of headaches per month using monthly migraine days (MMD).

### Treatment history

During the interview, patients’ medication histories, including both prophylactic and abortive treatments, were collected. Prophylactic treatment was defined as any specific or non-specific treatment to prevent migraines [3]. Prophylactic medication details were documented, including: (1) Type of medication, (2) Dose, (3) Duration of use (in months), (4) Frequency of administration per day, (5) The number of prophylactic treatments used included single drug or polytherapy which is defined as two or more drugs used, (6) Reported side effects, (7) Prescribing physician’s specialty, (8) Adherence to treatment and (9) Efficacy of prophylactic medication.

### Adherence to prophylactic treatment

Adherence to prophylactic treatment was defined as the extent to which patients follow their prescribed therapy over a fixed period [9]. Adherence was measured using the 12-item Arabic version of the Adherence to Refills and Medications Scale (ARMS), with a score of ≥ 16 used as the threshold for non-adherence and < 16 for adherence [10]. ARMS is a self-reported scale developed in English, consisting of two subscales: adherence to filling prescriptions and medication adherence [11].

### Headache-related disability

The Migraine Disability Assessment Scale (MIDAS) (Arabic version) [12] was used to assess the impact of migraines on daily activities before and after prophylactic treatment. MIDAS consists of five questions that evaluate the effect of headaches on three activity categories over the previous three months: Paid work (days off work and reduced efficiency), Household activities (days affected by headaches) and Recreational, social, and family events (missed days due to headaches) [13]. The total MIDAS score was calculated by adding the scores for each of the five questions.

#### Headache severity

Headache severity was assessed using the Visual Analogue Scale (VAS**)** before and after prophylactic treatment.

### Statistical analysis

Data were coded and maintained with the Statistical Package for the Social Sciences (SPSS) version 28 (IBM Corp., Armonk, NY, USA). Quantitative data was summarised using mean, standard deviation, median, minimum, and maximum, while categorical data was summarised using frequency (count) and relative frequency (%). The quantitative variables were compared using the non-parametric Kruskal-Wallis and Mann-Whitney tests. To compare serial measurements within each patient, the non-parametric Wilcoxon signed rank test was applied [14]. To compare categorical data, the Chi-square (χ²) test was used. An exact test is utilised when the anticipated frequency is fewer than five [15]. Non-parametrical methods were used as data deviated from the normal distribution, as noted by double-checking normality using normality tests and plots. Logistic regression was used to identify independent determinants of adherence [16]. P-values of less than 0.05 were considered statistically significant.

In our study, we used Jointpoint regression analysis to identify significant changes in trends within the data. The breakpoints were determined using a permutation test with a Monte Carlo stimulation approach to assess statistical significance. The Bayesian Information Criterion (BIC) and Goodness-of-Fit Statistics were used to select the optimal number of breakpoints, ensuring that the model balances complexity and explanatory power.

## Results

The baseline characteristics of the study population are displayed in Table 1. The median patient age was 32 years, and females accounted for 70.6% of the cohort. The median duration of disease was 16.5 months.

**Table 1:** Demographics and clinical characteristics of individuals with migraine

Item		Count
Age in years [median (IQR)]		32 (14–63)
Gender [n (%)]	Male	30 (29.4%)
	Female	72 (70.6%)
Education in years [median (IQR)]		16 (6–25)
Marital status [n (%)]	Single	32 (31.4%)
	Married	65 (63.7%)
	Divorced	5 (4.9%)
Employment status [n (%)]	Unemployed	49 (48.0%)
	Employed	53 (52.0%)
Comorbidities [n (%)]	Yes	29 (28.4%)
	No	73 (71.6%)
Specify comorbidity/s [n (%)]	Medical comorbidities	18 (62.1%)
	Psychiatric	11 (37.9%)
Health insurance [n (%)]	Yes	45 (44.1%)
	No	57 (55.9%)
Disease duration in months [median (IQR)]		16.5 (3-480)
Age at onset of migraine in years [median (IQR)]		18 (8–33)
Type of migraine [n (%)]	Episodic	54 (52.9%)
	Chronic	48 (47.1%)
Aura [n (%)]	Yes	31 (30.4%)
	No	71 (69.6%)
Type of aura [n (%)]	Visual	22 (71.0%)
	Sensory	9 (29.0%)

**Fig. 1:**
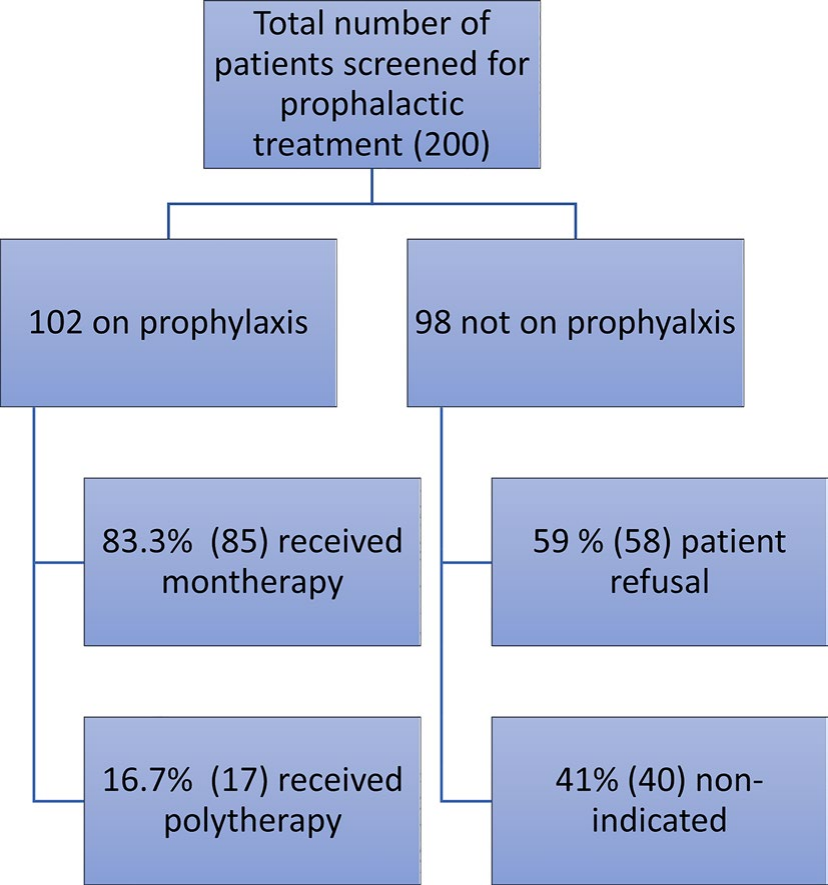
Analysis of the recruitted popultion

Of the 200 patients included in our study, 102 (51%) required prophylactic migraine treatment and were started on an appropriate regimen. The remaining 98 patients (49%) did not receive prophylactic therapy, either because it was not clinically indicated (40 patients) or because they declined treatment after discussing its potential benefits (58 patients), often citing prior negative experiences with prophylactic treatments or information obtained from social media. This finding highlights a gap in adherence and willingness to initiate therapy among eligible patients (Fig. 1).

The types of treatments (abortive or prophylactic) used in this cohort are presented in Table 2. Regarding prophylactic treatment, most of the patients (83.3%) received monotherapy; topiramate was the most prescribed prophylactic medication as monotherapy (25.49%). Antiepileptic drugs (AEDs) were used more in episodic migraine cases (P-value = 0.022), while polytherapy was more common in chronic migraine (CM) cases (P-value = 0.02) (Table 2).

**Table 2:** Types of migraine treatment used

Item		Total	Episodic migraine	Chronic migraine	*P* value
Acute treatment [n (%)]	Paracetamol	42 (41.2%)	27 (50%)	15 (31.3%)	< 0.001
	NSAID	23 (22.5%)	17 (31.5%)	6 (12.5%)	
	Triptan	29 (28.4%)	9 (16.7%)	20 (41.7%)	
	Polytherapy	8 (7.8%)	1 (1.9%)	7 (14.6%)	
Prophylactic treatment [n (%)]	BB	17 (16.7%)	11 (20.4%)	6 (12.5%)	0.022
	CCB	1 (1.0%)	1 (1.9%)	0 (0.0%)	
	AED	40 (39.2%)	27 (50%)	13 (27.1%)	
	Antidepressant	17 (16.7%)	7 (13.0%)	10 (20.8%)	
	Anti-CGRP	6 (5.9%)	2 (3.7%)	4 (8.3%)	
	Botox	3 (2.9%)	1 (1.9%)	2 (4.2%)	
	ARB	1 (1.0%)	1 (1.9%)	0 (0.0%)	
	Polytherapy	17 (16.7%)	4 (7.4%)	13 (27.1%)	

NSAID: nonsteroidal anti-inflammatory drugs, BB: betablocker, CCB: calcium channel blocker, AED: antiepileptic drugs, Anti-CGRP: calcitonin gene related peptide monoclonal antibodies ARBs: angiotensin receptor blockers. P-value > 0.05 is considered non-significant

Regarding acute treatment, paracetamol was the most commonly prescribed medication, used by 42% of patients. Nonsteroidal anti-inflammatory drugs (NSAIDs) were more frequently used in episodic migraine compared to chronic migraine, whereas triptans and combination therapy were more commonly utilized among chronic migraine individuals (*p* < 0.001) (Table 2).

**Fig. 2:**
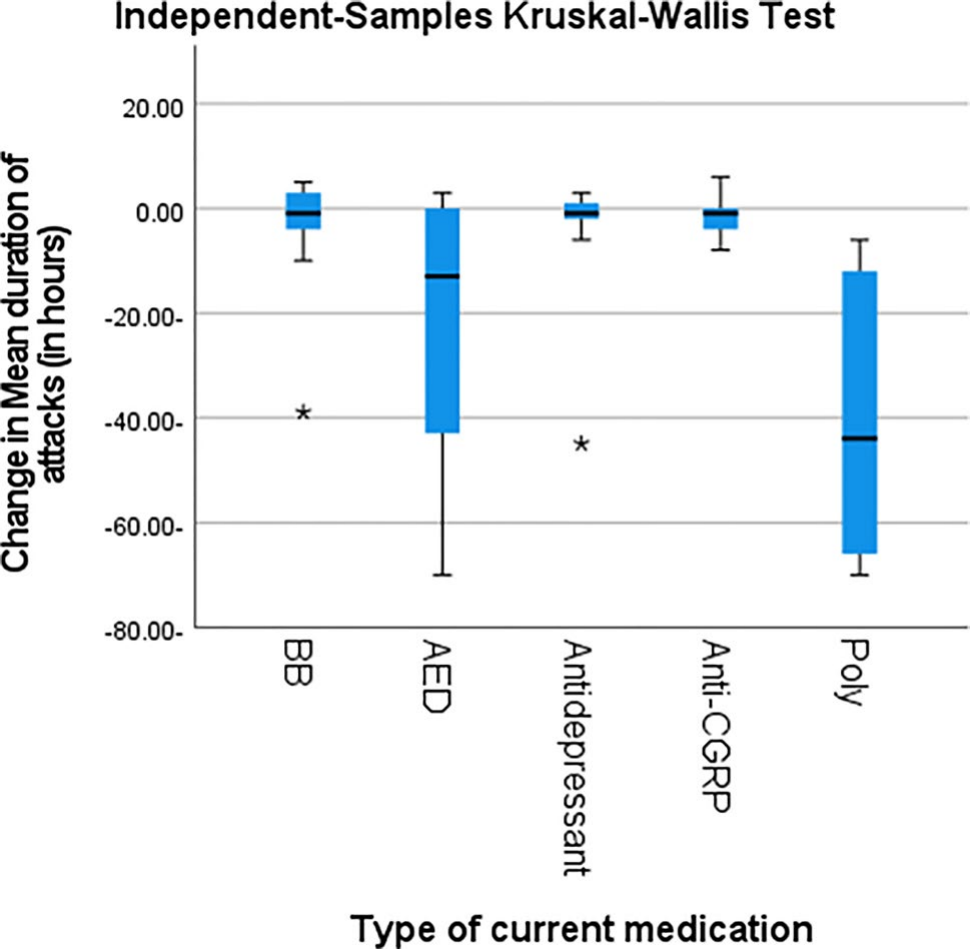
Post hoc pair wise comparisons among different used prophylactic drugs regarding the change in mean duration of attacks. BB: betablocker, AED: antiepileptic drugs, Anti-CGRP: calcitonin gene related peptide monoclonal antibodies Poly: polytherapy

**Fig. 3:**
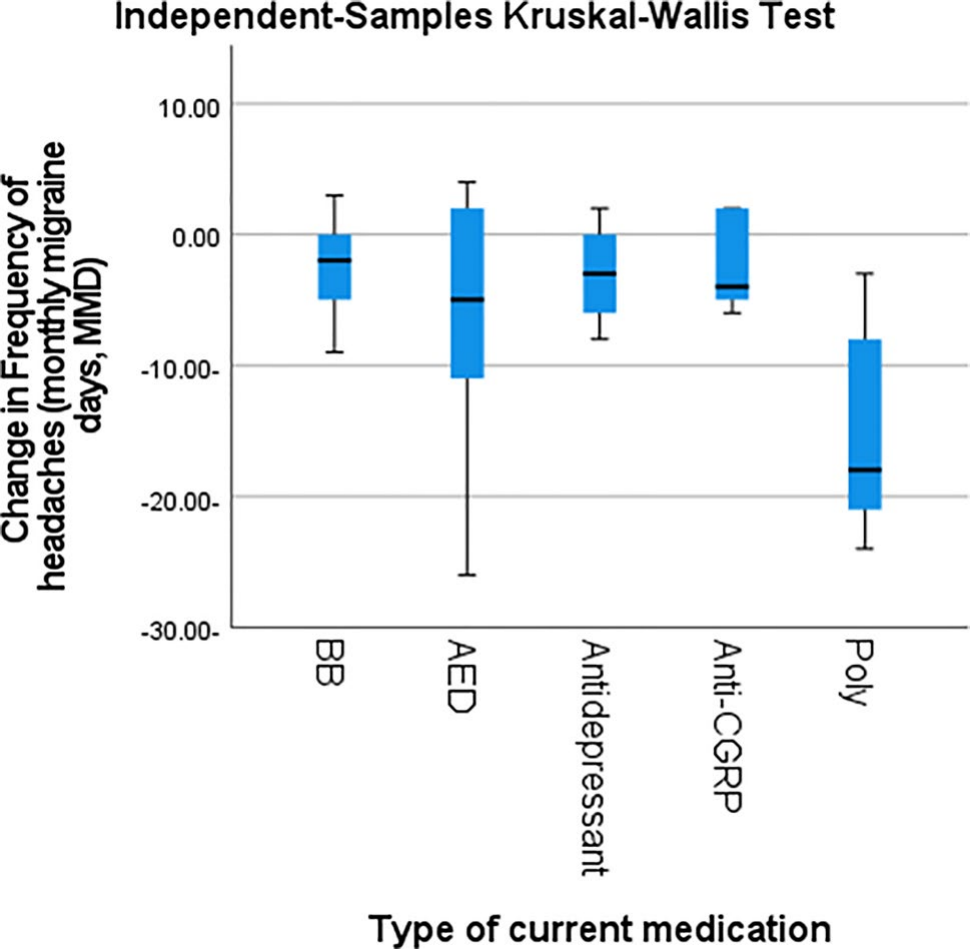
Post hoc pair wise comparisons among different used prophylactic drugs regarding the change in headcahes frequency. BB: betablocker, AED: antiepileptic drugs, Anti-CGRP: calcitonin gene related peptide monoclonal antibodies Poly: polytherapy, MMD: monthly migraine days

**Fig. 4:**
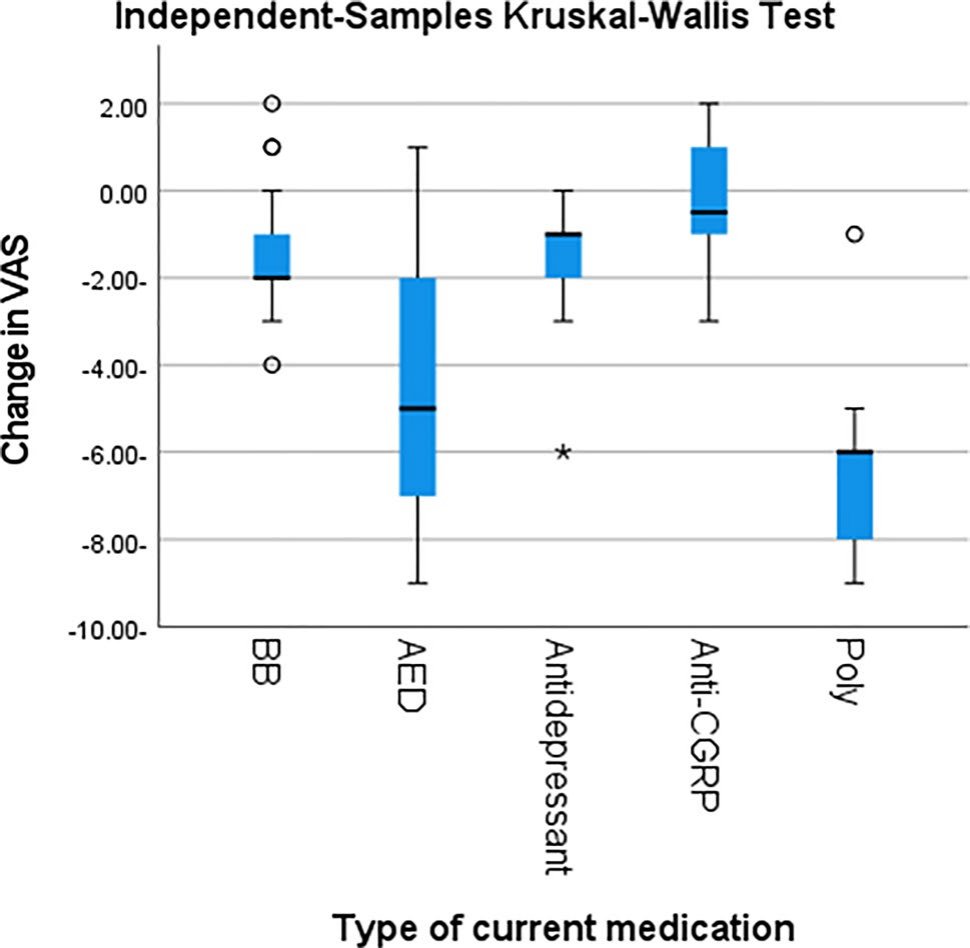
Post hoc pair wise comparisons among different used prophylactic drugs regarding the change in headcahes severity by VAS. BB: betablocker, AED: antiepileptic drugs, Anti-CGRP: calcitonin gene related peptide monoclonal antibodies, Poly: polytherapy, VAS: visual analogue scale

**Fig. 5:**
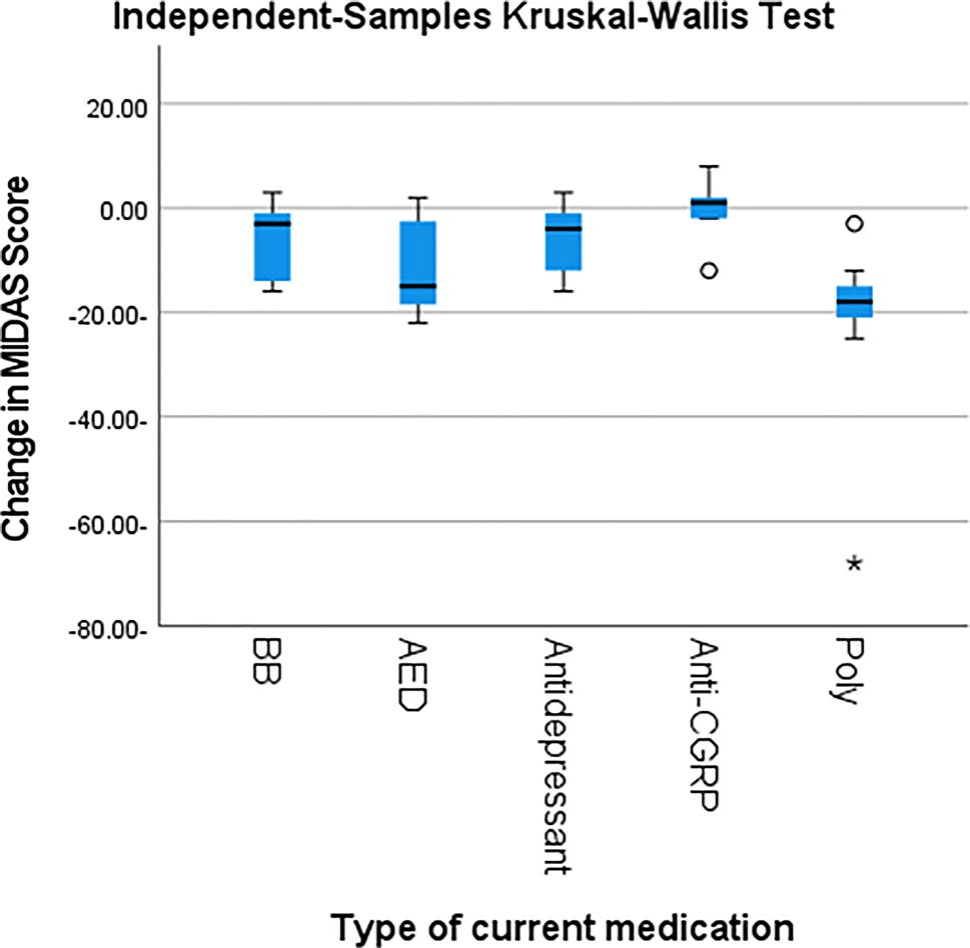
Post hoc pair wise comparisons among different used prophylactic drugs regarding the change in migraine disability by MIDAS. *BB: betablocker*,* AED: antiepileptic drugs*,* Anti-CGRP: calcitonin gene related peptide monoclonal antibodies* Poly: polytherapy, *MIDAS: Migraine Disability Assessment Scale*

Following prophylactic treatment, individuals with migraine demonstrated a significant reduction in attack duration, MMD, VAS scores, and MIDAS scores compared with baseline (*p* < 0.001) (Table 3) where polytherapy regimens followed by antiepileptic drugs (AEDs) were associated with significant reductions the above-mentioned parameters (*p* < 0.001) (Table 4; Figs. 2, 3, 4 and 5).

**Table 3:** Comparison between headache characteristics before and after treatment prophylactic treatments

	Mean	Standard Deviation	Median	Minimum	Maximum	*P* value
Mean duration of headache attacks (in hours) before treatment	20.86	23.03	9.50	2.00	72.00	< 0.001
Mean duration of headache attacks (in hours) After treatment	4.48	2.27	4.00	1.00	12.00	
Frequency of migraine headache attacks (monthly migraine days, MMD) before treatment	14.36	7.07	12.00	2.00	30.00	< 0.001
Frequency of migraine headache attacks (monthly migraine days, MMD) after treatment	8.18	5.94	8.50	1.00	22.00	
VAS (before treatment)	8.27	1.38	9.00	5.00	10.00	< 0.001
VAS (after treatment)	4.75	2.24	5.00	1.00	9.00	
MIDAS Score (before treatment)	18.09	8.14	20.00	4.00	68.00	< 0.001
MIDAS Score after treatment	7.57	5.16	8.00	0.00	24.00	

MMD: monthly migraine days, VAS: visual analogue scale, MIDAS: Migraine Disability Assessment Scale

P-value > 0.05 is considered non-significant

**Table 4:** Prophylactic treatment and characteristic headache in individuals with migraine

current medication		BB	AED	Antidepressant	Anti-CGRP	polytherapy	*P* value
Change in Mean duration of attacks (in hours)	Mean	-3.41	-21.75	-3.41	-1.33	-39.24	< 0.001
	SD	10.28	24.04	10.99	4.68	25.60	
	Median	-1.00	-13.00	-1.00	-1.00	-44.00	
	Minimum	-39.00	-70.00	-45.00	-8.00	-70.00	
	Maximum	5.00	3.00	3.00	6.00	-6.00	
Change in Frequency of headaches (monthly migraine days, MMD)	Mean	-2.24	-6.20	-2.65	-2.50	-14.88	< 0.001
	SD	3.44	7.72	3.35	3.62	7.22	
	Median	-2.00	-5.00	-3.00	-4.00	-18.00	
	Minimum	-9.00	-26.00	-8.00	-6.00	-24.00	
	Maximum	3.00	4.00	2.00	2.00	-3.00	
Change in VAS	Mean	-1.35	-4.43	-1.71	-0.33	-6.53	< 0.001
	Standard Deviation	1.62	2.95	1.36	1.75	1.97	
	Median	-2.00	-5.00	-1.00	-0.50	-6.00	
	Minimum	-4.00	-9.00	-6.00	-3.00	-9.00	
	Maximum	2.00	1.00	0.00	2.00	-1.00	
Change in MIDAS Score	Mean	-0.576	-11.48	-6.82	-0.33	-20.47	< 0.001
	Standard Deviation	6.91	8.08	6.55	6.62	13.37	
	Median	-3.00	-15.00	-4.00	1.00	-18.00	
	Minimum	-16.00	-22.00	-16.00	-12.00	-68.00	
	Maximum	3.00	2.00	3.00	8.00	-3.00	

MMD: monthly migraine days, VAS: visual analogue scale, MIDAS: Migraine Disability Assessment Scale, BB: betablocker, AED: antiepileptic drugs, Anti-CGRP: calcitonin gene related peptide monoclonal antibodies

P-value > 0.05 is considered non-significant

### Adherence to therapy

**Fig. 6:**
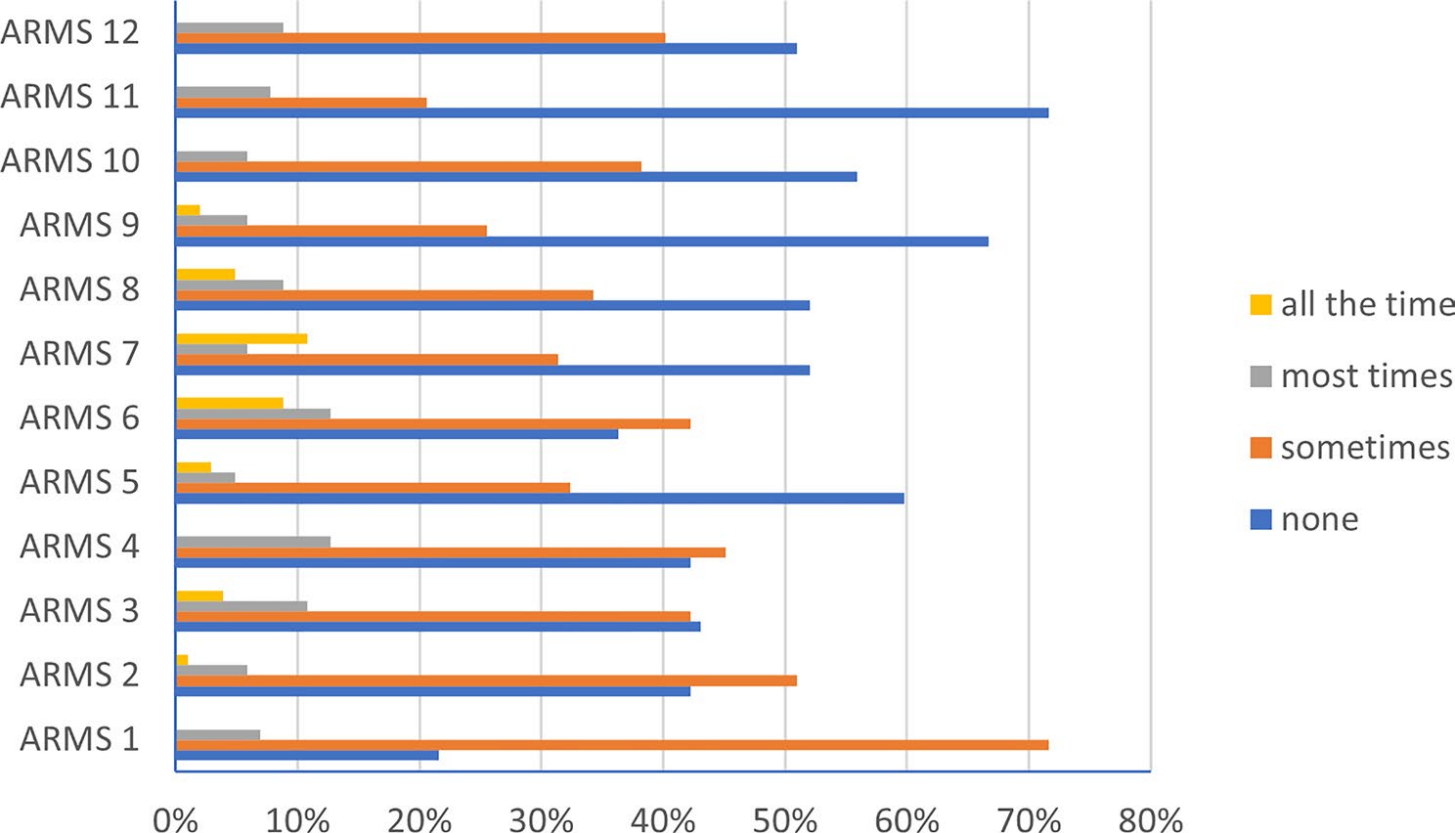
Analysis of Adherence to Refills and Medications Scale (ARMS) among studied population. ARMS: Adherence to Refills and Medications Scale, (1) How often do you forget to take your medicine?, (2) How often do you decide not to take your medicine?, (3) How often do you forget to get prescriptions filled?, (4) How often do you run out of medicine?, (5) How often do you skip a dose of your medicine before you go to the doctor?, (6) How often do you miss taking your medicine when you feel better?, (7) How often do you miss taking your medicine when you feel sick?, (8) How often do you miss taking your medicine when you are careless?, (9) How often do you change the dose of your medicines to suit your needs (like when you take more or less pills than you’re supposed to)?, (10) How often do you forget to take your medicine when you are supposed to take it more than once a day?, 11. How often do you put off refilling your medicines because they cost too much money?, 12. How often do you plan ahead and refill your medicines before they run out?

Adherence to prophylactic treatment was analyzed at least 3 months after drug initiation. During the follow-up period, 69.6% of patients were non-adherent to prophylactic treatment, whereas only 30.4% maintained adherence. In the Adherence to Refills and Medications Scale (Arabic version), the majority of responses to questions (Q) were “none of the time” (Q3: 43.1%, Q5: 59.8%, Q7: 52%, Q8: 52%, Q9: 66.7%, Q10: 55.9%, Q11: 71.6%, Q12: 51%), followed by “some of the time” (Q1: 71.6%, Q2: 51%, Q4: 45.1%, Q6: 42.2%) (Table 5; Fig. 6).

**Table 5:** Adherence to refills and medications scale (ARMS) in our study populations

	None of the time		Some of the time		most of the times		all of the time	
	Count	%	Count	%	Count	%	Count	%
ARMS 1	22	21.6%	73	71.6%	7	6.9%	0	0.0%
ARMS 2	43	42.2%	52	51.0%	6	5.9%	1	1.0%
ARMS 3	44	43.1%	43	42.2%	11	10.8%	4	3.9%
ARMS 4	43	42.2%	46	45.1%	13	12.7%	0	0.0%
ARMS 5	61	59.8%	33	32.4%	5	4.9%	3	2.9%
ARMS 6	37	36.3%	43	42.2%	13	12.7%	9	8.8%
ARMS 7	53	52.0%	32	31.4%	6	5.9%	11	10.8%
ARMS 8	53	52.0%	35	34.3%	9	8.8%	5	4.9%
ARMS 9	68	66.7%	26	25.5%	6	5.9%	2	2.0%
ARMS 10	57	55.9%	39	38.2%	6	5.9%	0	0.0%
ARMS 11	73	71.6%	21	20.6%	8	7.8%	0	0.0%
ARMS 12	52	51.0%	41	40.2%	9	8.8%	0	0.0%

ARMS: Adherence to Refills and Medications Scale, (1) How often do you forget to take your medicine?, (2) How often do you decide not to take your medicine?, (3) How often do you forget to get prescriptions filled?, (4) How often do you run out of medicine?, (5) How often do you skip a dose of your medicine before you go to the doctor?, (6) How often do you miss taking your medicine when you feel better?, (7) How often do you miss taking your medicine when you feel sick?, (8) How often do you miss taking your medicine when you are careless?, (9) How often do you change the dose of your medicines to suit your needs (like when you take more or less pills than you’re supposed to)?, (10) How often do you forget to take your medicine when you are supposed to take it more than once a day?, 11. How often do you put off refilling your medicines because they cost too much money?, 12. How often do you plan ahead and refill your medicines before they run out?

Adherent patients were more likely to be prescribed antidepressants (38.7% vs. 7% in the non-adherent group), whereas non-adherence was more frequent among those receiving antiepileptic drugs (46.5% vs. 22.6% adherent) or polytherapy (21.1% vs. 6.5% adherent) (Table 6).

**Table 6:** Comparison between adherent and nonadherent in patients on prophylactic treatment

			Adherent (*n* = 31)	Nonadherent (*n* = 71)	*P* value
Age	14–29 years		10 (32%)	32 (45.1%)	0.227
	30–63 years		21 (67.7%)	39 (54.9%)	
Gender [n (%)]		Male	11 (35.5%)	19 (26.8%)	0.374
		Female	20 (64.5%)	52 (73.2%)	
Marital status [n (%)]		Single	5 (16.1%)	27 (38%)	0.059
		Married	25 (80.6%)	40 (56.3%)	
		Divorced	1 (3.2%)	4 (5.6%)	
Education (median (IQR)) (in years)			16 (12–20)	16 (6–25)	0.610
Employment status [n (%)]		Unemployed	13 (41.9%)	36 (50.7%)	0.415
		Employed	18 (58.1%)	35 (49.3%)	
Comorbidities[n (%)]		Yes	10 (32.3%)	19 (26.8%)	0.571
		No	21 (67.7%)	52 (73.2%)	
Health inurance[n (%)]		Yes	15 (48.4%)	30 (42.3%)	0.566
		No	16 (51.6%)	41 (57.7%)	
Age at onset of migraine [median (IQR)] (in years)			20 (12–25)	18 (8–33)	0.038*
Type of migraine[n (%)]		Episodic	13 (41.9%)	41 (57.7%)	0.141
		Chronic	18 (58.1%)	30 (42.3%)	
Aura [n (%)]		Yes	11 (35.5%)	20 (28.2%)	0.460
		No	20 (64.5%)	51 (71.8%)	
Side Effect [n (%)]		Yes	8 (25.8%)	30 (42.3%)	0.114
		No	23 (74.2%)	41 (57.7%)	
MOH [n (%)]		Yes	10 (32.3%)	27 (38.0%)	0.577
		No	21 (67.7%)	44 (62%)	
Type of current medication [n (%)]		BB	7 (22.6%)	10 (14.1%)	< 0.001
		CCB	0 (0.0%)	1 (1.4%)	
		AED	7 (22.6%)	33 (46.5%)	
		antidepressant	12 (38.7%)	5 (7.0%)	
		Anti-CGRP	0 (0.0%)	6 (8.5%)	
		botox	2 (6.5%)	1 (1.4%)	
		ARB	1 (3.2%)	0 (0.0%)	
		polytherapy	2 (6.5%)	15 (21.1%)	

BB: betablocker, CCB: calcium channel blocker, AED: antiepileptic drugs, Anti-CGRP: calcitonin gene related peptide monoclonal antibodies ARBs: angiotensin receptor blockers, MOH: medication overuse headache

P-value > 0.05 is considered non-significant

Reported reasons for non-adherence included medication side effects (16.7%), perceived improvement in migraine severity and/or frequency (3.9%), cost of prophylactic treatment (2%), lack of efficacy (2%), prolonged duration of therapy (one patient), and influence from social media (one patient). Multivariate regression analysis was done to detect independent predictors of adherence. The independent variables were the use of antidepressants as prophylactic treatment (Table 7).

**Table 7:** Logistic regression to detect independent predictors of adherence

		*P* value	OR	95% C.I.	
				Lower	Upper
Adherence	Antidepressant	< 0.001	8.337	2.610	26.631

P-value > 0.05 is considered non-significant

### Polytherapy and migraine outcomes

Among patients receiving prophylactic treatment, 83.3% were on monotherapy, while 16.7% were on polytherapy regimens. Patients on polytherapy showed significantly greater reductions in headache duration, MMD, VAS scores, and MIDAS scores compared to those on monotherapy (*P* < 0.001) (Table 4). However, adherence rates were lower among patients receiving polytherapy (6.5%) than those on monotherapy (93.5%), indicating that while polytherapy may offer enhanced clinical benefits in certain patients, it is also associated with a higher likelihood of treatment discontinuation. The most frequently used prophylactic agents in polytherapy were AEDs combined with antidepressants or beta-blockers.

## Discussion

Migraines are a serious health and economic challenge [17]. Successful prophylactic treatment of migraine decreases disease burden, improves quality of life [18], and reduces the progression to chronic migraines [17]. However, preventative medicines are not particular to migraines because they are also used to treat depression, epilepsy, and hypertension. There has been evidence that these medications are frequently associated with low adherence to therapy, resulting in poor efficacy [2]. Therefore, it is essential to understand the current use of preventive medications in clinical practice. This represents the first Egyptian study investigating adherence patterns, clinical features and treatment patterns in individuals with migraine on prophylactic medications.

Despite the availability of multiple prophylactic treatment options, it was estimated that nearly 12–20% of patients with migraine use prophylaxis [19, 20], and around 38% of people with migraine could gain benefit from prophylactic treatment [17, 21]. In the current study, 49% of patients were not on prophylactic treatment. This was due to non-indication or refusal of patients to start even after explaining the value of prophylaxis due to previous experience with other patients and/or the effect of social media.

The current study confirmed that prophylactic treatment reduces attacks’ frequency, duration, and severity. Individuals with migraine had significantly lower duration, MMD, VAS, and MIDAS scores after using prophylactic treatment. Similarly, other studies confirmed the importance of prophylactic treatment despite its underuse in real-world clinical practice [2, 17, 22, 23].

The current study found that patients with migraine receiving preventive treatments had an inadequate level of adherence, with only 30.4% of patients adhering to their preventive medication. This agrees with previous research confirming the early therapeutic discontinuation trend and a low adherence rate. (26.2% in Orlando et al. [2], 37.8% Meyers et al. [5], 29% Berger et al. [24], and 29% in Hepp et al. [25]). Lafata et al. [4] showed considerably higher adherence rates in episodic migraine (EM) (56% at 12 months).

According to our study data, one reason for the patients’ non-adherence to prophylactic medication is that they had previously been prescribed prophylactic treatment but experienced no significant improvement. This was because 36% of the previous medication had been prescribed by a non-neurologist (otolaryngologist or internal medicine) who, unfortunately, prescribed non-approved AEDs for migraine prophylaxis (e.g. carbamazepine and oxcarbazepine) or the use of groups of drugs like muscle relaxants or gabapentin, or the use of approved medications but underdosed or prescribed for a short duration. Also, the lack of a clear plan and proper explanation of the expected efficacy, needed duration of treatment adherence to achieve appropriate response and possible adverse effects from prophylactic treatment by the treating physician renders the patients’ adherence. Anti-calitonin gene-related peptide monoclonal antibodies (CGRP mAbs) were only used in 6 (5.9%) of our patients, 4 of which had CM, as shown in Table 2. This low number is due to the high expenses of this group of drugs, which are scarcely used in Egypt except for only a small sector of insured patients, as Egypt is one of the low-middle income countries. Moreover, the high cost of prophylactic medications presents a significant challenge, particularly for uninsured patients, who may struggle to afford the long-term treatment required for optimal outcomes. This financial burden can contribute to reduced adherence to prescribed regimens, further complicating effective migraine management. Addressing these costs and improving access to affordable treatment options is essential, especially for patients in resource-constrained settings.

Regarding adherence to prophylactic treatment, our study found no statistically significant difference between episodic and chronic migraineurs. These results are unexpected, as we have better adherence among patients with CM than EM due to the greater disability and disease burden. In previous studies, it was found that patients with episodic migraine might be resistant to the concept of taking daily medication and might only prefer to take abortive treatment during their attacks [26]. On the other hand, CM is a more disabling disease. Patients may not experience this but, on the contrary, prefer using a treatment that reduces the frequency and severity of migraine attacks, which could lead to higher rates of adherence [25].

Gender and marital status did not show significant differences in adherence patterns. This is different from previous studies that showed more compliance in women than men due to more disabling and frequent attacks [27].

In agreement with previous studies [25; 28], this study showed that topiramate was the most prescribed prophylactic medication. This observation contrasts with a Japanese study [5], which found that calcium-channel blockers, followed by anticonvulsants, were the most often prescribed preventive drugs during the baseline period. This result is in line with recommendations from the Japan Headache Society for the management of CM [29]. However, in contrast to Berger et al. [24], who reported that antidepressants were the highest drug associated with non-adherence, our study found that antidepressants were more in the adherent group. The independent variable of adherence using regression analysis was the use of antidepressants as prophylactic treatment. Hepp and his colleagues suggested that many prophylactic treatments (amitriptyline, nortriptyline, gabapentin, and divalproex) were associated with significantly lower odds of adherence when compared to his reference prophylactic medication, topiramate [25]. However, Lafata et al. performed comparable research and observed that adherence did not differ significantly among the investigated treatments in their sample [4].

According to Silberstein, 2015, although monotherapy is favored, it frequently does not produce the required therapeutic outcome, and it may be needed to combine prophylactic medications.

Comorbidities must be considered because individuals with migraine frequently have coexisting medical and psychiatric conditions. However, using two distinct drugs may be necessary for the best treatment of a comorbid condition and migraine. Avoiding drug interactions or increased adverse events is a primary concern when using polypharmacy. Polytherapy may enable therapeutic adjustments based on the status of each illness [30]. This was in concordance with the present study, where the polytherapy regimen was the best regimen regarding MIDAS, VAS, MMD, and duration of the attacks (P-value < 0.001).

Combination therapies target several therapeutic pathways and are a reasonable and practical approach that may significantly enhance patient outcomes, especially for those with treatment-resistant migraine [31]. In this study polytherapy was more common in chronic migraine (CM) patients (P-value = 0.02).

In contrast to the present study, in which paracetamol was the most commonly prescribed abortive medication, in Japan, Meyers et al. found that triptans were the most common abortive medication received [5]. In contrast, in the United States of America (USA), Woolley et al. observed that opioids were the most prescribed acute medication [32]. In Italy, Orlando et al. found that the most common treatment regimen prescribed was NSAIDs [2]. This illustrates a significant difference in physician prescribing patterns for abortive medications in Egypt compared with the USA, countries in the European Union and Japan.

This study shows that some patients reported noncompliance due to the following reasons: medication side effects (16.7%), improvement of migraine attacks severity and/or frequency (3.9%), cost of prophylactic treatment (2%), inefficacy of treatment (2%), prolonged period on therapy (1 patient), and social media influence (1 patient). According to the analysis of the ARMS score, 8.8% of our study population missed taking their medication when they felt better, 10.8% when they felt sick, and 4.9% missed taking it because of a careless attitude. These barriers align with findings from previous studies, such as Smith et al. (2020) [33], who noted that medication-related side effects and patient misunderstanding of the benefits of treatment were primary contributors to low adherence rates among migraine sufferers. In contrast, studies like Johnson et al. (2018) [34] emphasized the role of healthcare provider-patient communication in improving adherence, suggesting that enhancing patient education could mitigate some of the barriers we observed.

Previous research suggests that low adherence may be attributable to several causes, including adverse effects and/or the lack of efficacy of prophylactic drugs [25, 34–38]. Even the most effective preventive medications reduce headache frequency by half in only 50% to 60% of patients who try them. Response to preventive medicines can be idiosyncratic, an expression of the pathophysiologic heterogeneity that likely underlies the phenotypic expression of migraine. The resulting trial and error process may frustrate patients looking for relief. Some patients may have difficulty with daily medication use. For some patients, starting preventive migraine treatment may be the first time they have had to acknowledge having a serious illness. For others, cost, access, or insurance status may be limiting factors [18]. As a result, proper selection must be made individually, considering other essential factors such as safety and efficacy. The treatment decision must ultimately lie between the physician and the patient [25].

The broader implications of our findings suggest that improving patient adherence significantly reduces migraine frequency and severity, ultimately improving patients’ quality of life. Future research should explore targeted interventions to address the identified barriers, such as simplifying treatment regimens and enhancing patient-provider communication to improve adherence and treatment outcomes.

Beyond individual-level factors, our findings should also be interpreted within the broader context of a low- and middle-income country (LMIC) setting. In Egypt, as in many LMICs, systemic barriers exert a profound influence on adherence to migraine prophylaxis. This includes frequent medication stockouts, high out-of-pocket costs even for generic drugs, and limited insurance coverage, all of which reduce access to consistent preventive therapy. Moreover, the shortage of neurologists and specialized headache clinics often results in insufficient patient education and inappropriate prescribing practices, as reflected in our finding that some patients had previously received suboptimal prophylactic regimens from non-specialists. In addition to structural barriers, sociocultural factors such as low health literacy, cultural stigma that trivializes migraine, and reliance on traditional remedies also hinder treatment uptake and adherence. These factors may drive patients toward repeated use of over-the-counter analgesics, perpetuating a cycle of poor adherence, inadequate control, and risk of medication-overuse headache. Addressing these challenges requires system-level interventions that go beyond patient-level strategies. Potential approaches include task-shifting to empower primary care providers in migraine management, securing reliable medication supply chains, improving insurance coverage, and implementing public awareness campaigns to reduce stigma and enhance health literacy.

## Limitations

This study has some limitations. First, the limited sample size hampered additional comparative analyses of migraine subgroups (adherent versus non-adherent patients). Second, the cross-sectional design made it impossible to determine the precise order in which medical events occurred. Also, some potential confounders, such as psychiatric comorbidities and socioeconomic status, were not explicitly controlled for in this study. Another limitation of our study is that while we described adherence patterns and clinical outcomes, we did not systematically evaluate socioeconomic determinants, healthcare system capacity, or sociocultural barriers that strongly influence adherence in LMICs. The absence of such data limits the ability to contextualize our findings within the full spectrum of challenges faced by patients in Egypt. Future research should therefore integrate socioeconomic indicators, drug availability, health literacy, and cultural perceptions to provide a more comprehensive understanding of adherence in LMIC contexts.

Although our study was conducted at two centers, both institutions are among the largest tertiary referral hospitals in Egypt, receiving patients from multiple governorates across the country. As such, the recruited participants represent a wide spectrum of socioeconomic backgrounds, urban and rural settings, and diverse demographic characteristics. This referral pattern strengthens the external validity of our findings and makes them broadly representative of the national population rather than a narrowly localized sample. It is recommended in future studies to have a larger cohort and a prospective design for more expression of adherence and causes of non-adherence and more generalizability of the results. Although the ARMS scale is validated for Arabic-speaking populations, it has not been explicitly validated for Egyptians. However, as per our experience in this study, it could be used and is still valid, reliable and representative. Also, one of the limitations is that polytherapy showed a higher response rate compared to monotherapy. Our study did not account for confounding factors such as migraine severity and duration, which are known to influence treatment outcomes. Further research incorporating these variables is needed to confirm these findings.

## Conclusion

Effective migraine management relies on adherence to prophylactic therapy, yet our study highlights the importance of tailored treatment strategies to improve adherence and outcomes. Clinicians should prioritize patient education, address side effects proactively, and set realistic expectations. Ensuring proper dosing and follow-up care is essential, particularly in low-resource settings.

## Electronic Supplementary Material

Below is the link to the electronic supplementary material.

Supplementary Material 1

## Data Availability

Authors report that the datasets used and/or analyzed during the current study are available from the corresponding author upon reasonable request.

## Abbreviations

ARMS: Adherence to Refills and Medications Scale
MMD: Monthly migraine days
MIDAS: Migraine Disability Assessment Scale
VAS: Visual analogue scale
ICHD-3 criteria: The International Classification of Headache Disorders 3rd edition
SPSS: Statistical Package for the Social Sciences
NSAIDs: Nonsteroidal anti-inflammatory drug
AED: Antiepileptic drugs
Anti-CGRP: Calcitonin gene-related peptide antibodies
CM: Chronic migraine
EM: Episodic migraine
USA: United States of America
EU: European Union
