# Supplementary material for: Real-world assessment of current migraine prophylaxis in Egypt: a multicenter national study
Source: J Headache Pain. 2026 Jan 8;27(1):12. doi: 10.1186/s10194-025-02246-2 (PMC12784583; doi:10.1186/s10194-025-02246-2)
Supplement: Supplementary file 1 — Supplementary Material 1 [file 10194_2025_2246_MOESM1_ESM.docx]

# STROBE Flow Diagram

Figure 1: STROBE flow diagram illustrating patient screening, exclusions, and inclusion in the study.

**Patients screened for eligibility**

**(n=1100)**

**Received prophylaxis** (n=102)

Analyzed for adherence, efficacy and treatment patterns

**Did not receive prophylaxis (n=98)**

- Not clinically indicated (n=40)
- Declined treatment (n=58)

Eligible and included (n=200)

Excluded (n=900)
